# Opioid-free anesthesia in enhanced recovery after supratentorial craniotomies: a case series

**DOI:** 10.25122/jml-2024-0280

**Published:** 2024-12

**Authors:** Irwan Setiadi, Muhammad Rezanda Alifahna, Radian Ahmad Halimi, Dewi Yulianti Bisri

**Affiliations:** 1 Department of Anesthesiology and Intensive Care, Faculty of Medicine Universitas Padjadjaran Bandung, Indonesia; 2 Department of Anesthesiology and Intensive Care, Dr. Hasan Sadikin National Referral and Teaching Hospital, Bandung, Indonesia

**Keywords:** craniotomy, ERAS, opioid-free, perioperative analgesia, anesthesia

## Abstract

Enhanced Recovery After Surgery (ERAS) is a recovery method developed to minimize pain and improve post-operative healing in patients. Brain tumor resection using the ERAS concept is relatively new. This case series evaluates the implementation of the ERAS protocol in three female patients diagnosed with supratentorial brain tumors. All patients presented with headache as a primary symptom and underwent craniotomy under general anesthesia supplemented with a scalp block. The surgeries were performed with stable hemodynamics, and anesthesia was maintained using dexmedetomidine, propofol, and rocuronium. After surgery, the patients were immediately extubated and treated in the intensive care unit. The use of opioid-free anesthesia significantly supported the ERAS principles by reducing pain, postoperative nausea and vomiting (PONV), and shivering. This case series highlights the potential of opioid-free anesthesia as an effective alternative to opioids, aligning with the ERAS protocols to improve postoperative outcomes in brain tumor resection.

## INTRODUCTION

Enhanced Recovery After Surgery (ERAS) is a recovery method developed to minimize pain and improve post-operative healing in patients. The ERAS concept in neurosurgical procedures is a relatively recent development to reduce hospitalization duration [1]. Various components within the ERAS concept, including pain management during and after surgery, have been proven to provide long-term benefits for patients. The current pattern of pain management in patients undergoing neurosurgical operations is increasingly shifting to the concept of using non-opioid agents [1,2].

Brain tumors are abnormal intracranial growths of cell masses in the supratentorial and infratentorial compartments. Brain tumors are classified into primary and secondary (metastatic) tumors [3]. According to the American Cancer Society, approximately 24,530 cases of brain and nervous system tumors were diagnosed in 2021, with an estimated annual incidence rate of 7‒19.1 cases per 100,000 people [3,4]. Surgical intervention of supratentorial tumors aims to improve a patient's quality of life by addressing neurological deficits and alleviating symptoms such as pain and weakness. The rising incidence of such tumors underscores the need for innovative approaches, such as ERAS, to promote early postoperative recovery while reducing the duration and costs of hospital care [5].

Long-term opioid use, starting with acute pain management during the intraoperative and postoperative periods, often fails to deliver the expected benefits and is associated with a significant risk of opioid misuse. In addition, patients who have never received opioid treatment are more likely to repeat postoperative opioid administration, which is associated with a large increase in opioid misuse [6]. This finding is also confirmed by other studies that found opioid misuse due to uncontrolled use of opioids when treating acute postoperative pain [7]. This case series explores the integration of opioid-free anesthesia into the ERAS protocol for supratentorial brain tumor surgeries.

## CASE ILLUSTRATIONS

### Case 1: Supratentorial tumor with visual disturbances

#### Patient history

A 35-year-old female (body weight: 60 kg, height: 160 cm) presented with complaints of headaches and progressive blurry vision in the right eye, which became increasingly worse over time. She denied nausea, vomiting, and seizures. The patient had a two-year history of hypertension managed with amlodipine (10 mg daily).

#### Clinical examination

The patient was alert and fully oriented (Glasgow Coma Scale [GCS] score: 15). Vital signs were stable: blood pressure 147/92 mmHg, pulse 78 bpm, respiratory rate 18/min, temperature 36.4°C, and oxygen saturation 98% on room air. Neurological evaluation revealed isochoric pupils and reduced visual acuity (4/60 in the right eye and >6/60 in the left eye). No neck stiffness, positive Lasegue, Kernig signs, or hemiparesis were observed.

#### Diagnostic findings

**Figure 1:**
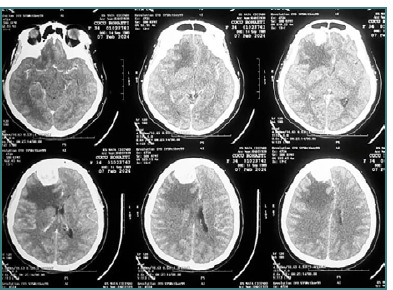
CT scan shows compressed sulcus and gyrus, compressed Sylvian fissure, peritumoral edema, and midline shift

Laboratory investigations and chest X-rays were within normal limits. A CT scan showed a compressed sulcus and gyrus, compressed Sylvian fissure, peritumoral edema, and a midline shift of 9.22 mm (Figure 1). The patient was diagnosed with a supratentorial space-occupying lesion (SOL) in the right frontal region, with differential diagnoses including a convexity meningioma or a frontal base meningioma. The patient was scheduled to undergo craniotomy for tumor removal.

#### Preoperative management

**Figure 2:**
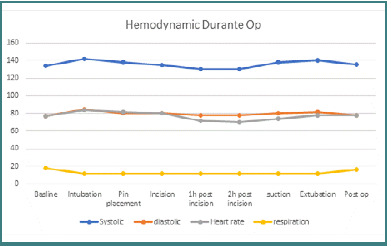
Graph shows stable hemodynamics

The patient was counseled regarding the planned anesthesia and surgery, and informed consent was obtained. An 18G intravenous line was inserted, and preoperative fasting guidelines were followed (8 hours for solids and 2 hours for liquids). Amlodipine was administered to control blood pressure (Figure 2).

#### Anesthesia management

Two hours before induction, the patient received intravenous paracetamol (1 g). Induction was achieved using dexmedetomidine (loading dose: 60 mcg over 10 minutes, maintenance: 0.2-0.7 mcg/kg/hour), propofol (150 mg), rocuronium (50 mg), and lidocaine (60 mg). Intubation was performed using a size 7.0 endotracheal tube. Maintenance anesthesia included dexmedetomidine (0.2–0.6 mcg/kg/hour), propofol (50–150 mcg/kg/min), and intermittent rocuronium (0.15 mg/kg every 45 minutes). Additional measures included intravenous dexamethasone (10 mg), a scalp block with 0.25% bupivacaine, arterial line placement, and central venous catheterization. Mannitol (1 g/kg) was administered before dural incision.

At the end of the operation, after spontaneous breathing was obtained, the patient was given a muscle relaxant reversal agent. Maintenance propofol was stopped while dexmedetomidine was continued at a dose of 0.2 mcg/kg/hour. The patient was extubated, fully conscious, and pain-free (VAS 1/10).

#### Post-surgical management

After surgery, the patient was taken to the intensive care unit (ICU) fully conscious and breathing spontaneously. A neurological assessment was conducted immediately. Postoperative analgesia included intravenous paracetamol (1 g every 6 hours) and continued dexmedetomidine infusion (0.2 mcg/kg/hour) for 24 hours. The patient did not report any complaints of pain (maximum VAS 1/10), nausea, vomiting, shivering, or respiratory depression during the postoperative period. She was discharged from the ICU after one day and sent home on the third postoperative day.

### Case 2: Supratentorial tumor with ocular protrusion

#### Patient history

A 43-year-old female (body weight: 50 kg, height: 158 cm) presented with headaches and progressive left-eye protrusion. The patient denied complaints of nausea, vomiting, and seizures. Her medical history included a five-year history of hypertension, managed with amlodipine (5 mg daily).

#### Clinical examination

The patient was alert and oriented (GCS score: 15). Vital signs were stable: blood pressure 135/75 mmHg, pulse 85 bpm, respiratory rate 20/min, temperature 36.4°C, and oxygen saturation 98% on room air. Neurological examination revealed anisocoric round pupils and reduced visual acuity (right eye: >6/60, left eye: 4/60). Signs of meningeal irritation were absent, with no neck stiffness or abnormal Lasegue or Kernig signs. No hemiparesis was observed.

#### Diagnostic findings

**Figure 3:**
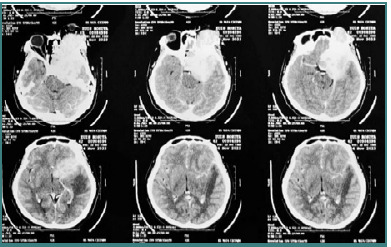
CT scan showed that the sulcus and gyri were not compressed, there was no peritumoral edema and midline shift

Laboratory investigations and chest X-ray results were within normal limits. CT imaging showed no sulcal or gyral compression, peritumoral edema, or midline shift (Figure 3). The patient was diagnosed with a supratentorial space-occupying lesion in the left sphenoorbital region extending into the left temporal, left frontal, sellar, left para sellar, left retrobulbar, and left cavernous regions. The lesion was suspected to be a meningioma. The patient was scheduled to undergo craniotomy for tumor removal.

#### Preoperative management

**Figure 4:**
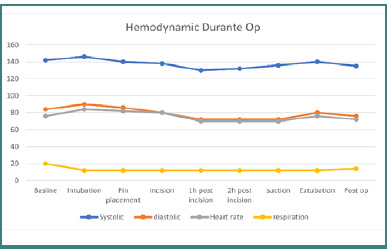
Intraoperative hemodynamic stability

The patient was provided a detailed explanation of the anesthesia procedure and signed informed consent. An 18G intravenous line was established, and fasting guidelines were followed. Amlodipine was administered as per her regimen (Figure 4).

#### Anesthesia management

The patient received intravenous paracetamol (1 g) two hours before induction. Induction involved dexmedetomidine (loading dose: 50 mcg over 10 minutes, maintenance: 0.2-0.7 mcg/kg/hour), propofol (120 mg), rocuronium (40 mg), and lidocaine (50 mg). A 7.0 endotracheal tube was used for intubation. Anesthesia was maintained with dexmedetomidine (0.2-0.7 mcg/kg/hour), propofol (50-150 mcg/kg/minute), and intermittent rocuronium (0.15mg/kg every 45 minutes). After induction, the patient was given dexamethasone (10 mg) and underwent a scalp block with 0.25% bupivacaine, placement of an arterial line, and central venous catheter (CVC) access. Mannitol solution (1g/kg) was given before the dural incision.

At the end of the procedure, once spontaneous breathing was confirmed, a muscle relaxant reversal agent was administered, and propofol infusion was stopped while dexmedetomidine was maintained at 0.2 mcg/kg/hour. The patient was extubated, fully conscious, and pain-free (VAS 1/10).

#### Post-surgical management

The patient was transferred to the ICU, fully conscious and breathing spontaneously. A comprehensive neurological examination was promptly conducted upon ICU admission. Postoperative analgesia included intravenous paracetamol (1 g every 6 hours) and continued dexmedetomidine infusion (0.2 mcg/kg/hour) for 24 hours. The patient had no complaints of pain (maximum VAS 2/10), nausea, vomiting, shivering, or respiratory depression during the postoperative period. After 1 day of ICU treatment, the patient returned to the room and went home after 2 days of treatment in a surgical ward.

### Case 3: Supratentorial tumor with hemiparesis

#### Patient history

A 46-year-old female (body weight: 58 kg, height: 154 cm) presented with a four-month history of progressive right limb weakness. Complaints were accompanied by recurring and worsening headaches. The patient denied complaints of nausea and vomiting. A history of two seizure episodes one month prior was noted, with the patient regaining full consciousness after each event.

#### Clinical examination

**Figure 5:**
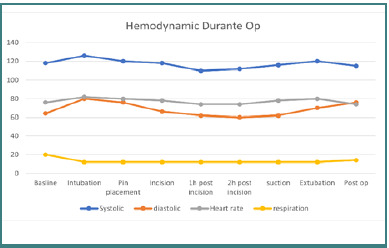
Graph shows stable hemodynamics.

The patient was alert and oriented (GCS score: 15). Vital signs were stable: blood pressure 112/83 mmHg, pulse 88 bpm, respiratory rate 18/min, temperature 36.4°C, and oxygen saturation 98% on room air (Figure 5). A neurological examination revealed hemiparesis in the right extremities. No neck stiffness, positive Lasegue, Kernig signs, or other focal deficits were noted.

#### Diagnostic findings

**Figure 6:**
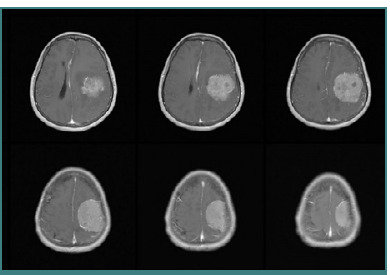
CT scan showed compressed sulcus and gyrus, peritumoral edema, midline shift

Laboratory and chest X-ray results were within normal limits. The CT scan showed that the sulcus and gyri were compressed, evidence of peritumoral edema, and a significant midline shift (Figure 6). The patient was diagnosed with a supratentorial space-occupying lesion in the left frontoparietal region, likely a convexity meningioma. The patient was scheduled for craniotomy and tumor resection.

#### Preoperative management

The patient was provided a detailed explanation of the anesthesia procedure and signed informed consent. An 18G intravenous line was established, and preoperative fasting protocols were adhered to—solid foods were restricted for 8 hours, while clear liquids were allowed up to 2 hours before surgery. The patient was administered amlodipine as scheduled for blood pressure regulation.

#### Anesthesia management

The patient received intravenous paracetamol (1 g) two hours prior to induction. Anesthesia induction was achieved using dexmedetomidine (1 mcg/kg over 10 minutes, followed by a maintenance dose of 0.2-0.6 mcg/kg/hour), propofol (2.5 mg/kg), rocuronium (0.8 mg/kg), and lidocaine (1 mg/kg). Intubation was performed with a size 7.0 endotracheal tube. Maintenance anesthesia included dexmedetomidine (0.2-0.6 mcg/kg/hour), propofol (50-150 mcg/kg/minute), and intermittent rocuronium (0.15 mg/kg every 45 minutes). Following induction, the patient received intravenous dexamethasone (10 mg) and a scalp block with 0.25% bupivacaine. An arterial line and central venous catheter (CVC) were placed, and mannitol (1 g/kg) was administered intravenously before the dura mater incision.

At the end of the surgery, spontaneous breathing was confirmed, and a muscle relaxant reversal agent was administered. Propofol infusion was discontinued, while dexmedetomidine was continued at 0.2 mcg/kg/hour. The patient was admitted to the ICU and, after extubation, was found to be fully conscious and pain-free (VAS 1/10). Following one day of ICU care, the patient was transferred to the general ward and discharged home after two days of recovery in the surgical ward.

## DISCUSSION

ERAS is a standardized and multidisciplinary perioperative care protocol that minimizes perioperative stress and improves surgical outcomes [1,8]. The application of ERAS in craniotomy for tumor resection is a relatively novel concept, particularly in neurosurgical procedures, where perioperative analgesia plays a central role in its success [1]. One of the primary challenges faced by clinicians is achieving adequate analgesia after craniotomy while maintaining cognitive function and promoting early mobilization, key components of ERAS [1]. According to Liu *et al*., mild and well-tolerated postoperative pain after elective craniotomy significantly enhances patient satisfaction and enables early mobilization, reducing ICU stay and overall hospitalization duration [9].

The aim of selecting analgesics in patients undergoing surgery for intracranial tumors is to control perioperative pain with drugs that have minimal effects on cognitive function and orientation. Therefore, the administration of non-opioid agents is expected to be able to replace the role of opioids as an adequate analgesic in intra-operative neurosurgery [10]. A study by Darmawikarta *et al*. highlighted that the use of non-opioid analgesics, such as regional anesthesia (e.g., scalp block) combined with sedative-hypnotic agents like dexmedetomidine or propofol, can effectively replace opioids in supratentorial tumor resection [11]. However, research comparing opioid-free approaches in intracranial tumor surgery is still very limited [10,11].

Dexmedetomidine is an alpha-2 agonist which has hypnotic, sedative, and analgesic effects [12]. Studies demonstrate its ability to reduce postoperative opioid use by approximately 60% while also stabilizing perioperative hemodynamics through decreased plasma catecholamine levels during surgery. Dexmedetomidine has demonstrated analgesic effects without significant respiratory depression, as it provides good perioperative hemodynamic stability with reduced intraoperative opioid requirements. According to a randomized control trial study by Tanskanen *et al*., dexmedetomidine demonstrated improved perioperative hemodynamic stability in patients undergoing brain tumor surgery. Compared with fentanyl, the trachea is intubated more quickly without respiratory depression [13]. Another study, a randomized controlled trial by Batra *et al*., showed that dexmedetomidine infusion initiated preoperatively maintained intraoperative hemodynamic stability and attenuated the cardiovascular response to intubation, skull pin placement, and extubation. In addition, dexmedetomidine also reduces the need for other anesthetic agents [14].

'Scalp block' is a regional anesthesia technique that targets peripheral nerve fibers of the scalp, such as the supraorbital, supratrochlear, temporal zygomatic, auriculotemporal, greater occipital, and lesser occipital nerves [15]. Scalp block can be performed for all supratentorial intracranial procedures and can minimize the hemodynamic response to surgical stimuli, reduce the need for intraoperative anesthesia, reduce postoperative pain, and reduce morphine analgesia. In intracranial tumor surgery, scalp block prevents hemodynamic changes during scalp incision [15,16]. Hemodynamic instability can lead to increased cerebral blood flow, exacerbating brain edema, or raising ICP, which can be detrimental in neurosurgical settings [15–17].

In this case series, we presented three cases of patients who underwent supratentorial tumor resection craniotomy. Perioperative pain management incorporated a multimodal approach, avoiding opioids and utilizing agents such as paracetamol, dexmedetomidine, propofol, rocuronium, and lidocaine. Two hours prior to induction, patients were administered intravenous paracetamol (1 g). Induction was achieved with dexmedetomidine, propofol, rocuronium, and lidocaine, followed by maintenance anesthesia with dexmedetomidine and propofol. After spontaneous breathing was obtained, patients were given a muscle relaxant reversal agent at the end of the operation. In neurosurgical operations, intravenous anesthesia techniques are often preferred over inhalation anesthesia. The combination of dexmedetomidine and propofol proved effective in replacing the role of opioids, providing adequate intraoperative analgesia and hemodynamic stability [17,18].

In this case series, there were no significant hemodynamic fluctuations. In addition, anesthetic induction was administered slowly to avoid large hemodynamic fluctuations and keep autoregulation within normal limits. This condition can occur if the average arterial pressure is 50‒150 mmHg. Mean arterial pressure below 50 mmHg can cause ischemia in brain tissue, while pressure above 150 mmHg will cause damage to the blood-brain barrier, resulting in brain edema or bleeding [18]. Intraoperative anesthesia management aims to avoid an increase in ICP brain edema and prevent secondary brain injury. Factors to avoid intraoperatively include hypoxemia, hypercapnia, anemia, and hypotension. In addition, brain autoregulation and response to CO2 levels also need to be maintained. Sedation can also cause the risk of hypercapnia, hypoxemia, and partial airway obstruction, thereby exacerbating the increase in ICP [6].

Pain experienced during or after tumor resection craniotomy is generally not a major concern, as the level of pain is typically considered tolerable [12]. Acute pain after craniotomy is believed to be less intense compared to other surgical procedures [12]. In theory, acute pain tends to be lower due to fewer pain receptors in the dura, pain insensitivity in the brain, reduced pain fiber density along the surgical incision line, and the development of auto analgesia. If analgesic therapy is inadequate, patients may experience pain, especially in the first hour after surgery, which may continue until the first or second day after surgery [12,19]. In this case series, all three patients reported minimal pain after craniotomy surgery.

One common postoperative complication is postoperative nausea and vomiting (PONV), which occurs in approximately 47% of post-craniotomy patients. PONV poses significant risks, such as increased ICP, intracranial hemorrhage, brain edema, and aspiration. The incidence of PONV often causes discomfort rather than postoperative pain. In a preoperative patient survey, vomiting was ranked as the most undesirable postoperative outcome, followed by pain and nausea. Although PONV rarely results in life-threatening conditions, it significantly affects patient satisfaction and comfort [20]. Serotonin antagonists and corticosteroids are useful in suppressing PONV [21]. In this case series, all three patients received dexamethasone postoperatively and reported no episodes of nausea or vomiting, which facilitated early mobilization within a day after surgery (Table 1). Adequate pain management and control of PONV allow ambulation as early as the first postoperative day in neurosurgical patients, facilitating early mobilization. Early mobilization is beneficial in preventing venous thrombosis, reducing muscle mass, reducing infection rates, and shortening the duration of treatment [21].

In this case series, there was no respiratory depression. In this case series, there were no instances of respiratory depression. Continuous administration of dexmedetomidine, known for its opioid-sparing effect, effectively reduces the minimum alveolar concentration (MAC) of anesthetic agents [12]. Dexmedetomidine is an effective sedative with minimal respiratory depression, ensuring airway patency and stable hemodynamics. However, caution is necessary when using untitrated doses, as they can result in hypotension, bradycardia, or post-anesthesia delirium [12].

**Table 1:** Follow up of side effects after craniotomy surgery.

	1^st^ patient				2^nd^ patient				3^rd^ patient			
	Nausea	Vomiting	Shivering	Pain	Nausea	Vomiting	Shivering	Pain	Nausea	Vomiting	Shivering	Pain
6 post-op hours	No	No	No	1	No	No	No	2	No	No	No	1
12 post-op hours	No	No	No	1	No	No	No	1	No	No	No	1
18 post-op hours	No	No	No	1	No	No	No	1	No	No	No	1
24 post-op hours	No	No	No	0	No	No	No	1	No	No	No	1
48 post-op hours	No	No	No	0	No	No	No	1	No	No	No	0
72 post-op hours	No	No	No	0	No	No	No	1	No	No	No	0

ERAS protocols accelerate recovery, reduce hospitalization duration, and lower treatment costs. However, managing perioperative pain during craniotomy remains a significant challenge for anesthesiologists, particularly with the growing application of ERAS in neurosurgical procedures, including tumor resection craniotomy [22]. Comprehensive preoperative preparation is essential to facilitate early postoperative recovery. This includes patient counseling, optimizing comorbid conditions, ensuring appropriate fasting, and administering antimicrobial prophylaxis. Additionally, prompt and thorough postoperative evaluation by neurosurgery clinicians is critical to support ERAS implementation. Opioid-free anesthesia plays a key role in ERAS by reducing pain, minimizing the incidence of PONV and shivering, and enhancing overall recovery. The successful application of ERAS in neurosurgical patients demonstrates its practicality and significant benefits [21,22].

## CONCLUSION

In this case series, opioid-free anesthesia effectively replaced opioids as the primary analgesic for perioperative pain management in tumor resection craniotomy surgery. This approach also minimized the risk of postoperative side effects, such as respiratory depression and PONV. Using opioid-free anesthesia proved to be a valuable strategy in supporting the ERAS concept.
